# Cardiac Manifestations of Sjogren's Syndrome: A Review of Literature

**DOI:** 10.7759/cureus.41002

**Published:** 2023-06-26

**Authors:** Larabe Farrukh, Aqsa Mumtaz, Sumbal Wajid, Hafiza Hareem Waqar, Ruben Peredo-Wende

**Affiliations:** 1 Internal Medicine, Albany Medical Center, Albany, USA; 2 Internal Medicine, Montefiore St. Luke's Cornwall Hospital, Newburgh, USA; 3 Rheumatology, Albany Stratton VA (Veteran Affairs) Medical Center, Albany, USA

**Keywords:** atrioventricular heart block, adults, autoimmune disease, cardiac manifestation, primary sjogren syndrome (pss)

## Abstract

Sjogren's syndrome (SS) is a chronic inflammatory disorder of the exocrine glands. It is characterized by a lymphocytic infiltrate in the lacrimal and salivary glands causing keratoconjunctivitis sicca and xerostomia. Extra-glandular involvement may be present in about one-third of patients with primary Sjogren’s syndrome (pSS). The most commonly affected organs are the thyroid, lungs, gastrointestinal tract, kidneys, skin, and nervous system. Cardiac manifestations of Sjogren's syndrome are rare and not well-described in the current literature. Most of the evidence is present in the form of case reports and small case series. However, recent studies have shown that patients with Sjogren's syndrome (SS) seem to have a greater overall risk of cardiovascular (CV) events. Although not conventionally considered a feature of the disease, cardiac manifestations can lead to increased morbidity and mortality in this patient population. In this review article, we study the association between cardiac diseases and primary Sjogren's syndrome.

## Introduction and background

Sjogren's syndrome (SS) is a chronic multisystem autoimmune disease that mainly affects the exocrine glands. It is characterized by a lymphocytic infiltrate in the lacrimal and salivary glands causing dry eyes (keratoconjunctivitis sicca) and dry mouth (xerostomia). Sjogren's syndrome is divided into two types, primary and secondary. Primary Sjogren's syndrome (pSS) occurs in the absence of any other rheumatologic disease while secondary Sjogren's syndrome occurs in conjunction with other autoimmune diseases like rheumatoid arthritis (RA) and systemic lupus erythematosus (SLE). Extra-glandular involvement may be present in about one-third of patients with primary Sjogren’s syndrome (pSS). The most affected organs are the thyroid, lungs, gastrointestinal tract, blood, kidneys, skin, and the central and peripheral nervous system. The involvement of the cardiovascular system is rare and is thought to be the consequence of multiple factors, including genetics, autoimmunity, and chronic inflammatory processes. While cardiac manifestations in SLE and rheumatoid arthritis are well studied, the literature on these cardiac manifestations in primary Sjogren's syndrome remains limited. As per anecdotal evidence, there have been reports of involvement of the cardiac conduction system, pericardium, myocardium, cardiac valves, and cardiac vasculature in primary Sjogren's syndrome. In this article, we aim to study the array of cardiac manifestations that have been reported in patients with primary Sjogren's syndrome.

## Review

Objectives 

In this article, we will discuss the various types of rare cardiac presentations in patients with primary Sjogren’s syndrome. 

Methods

A literature search was conducted on PubMed, Cochrane, and Google Scholar from 1990 to February 2023, using MeSH terms for Sjogren's syndrome combined with cardiac defects and manifestations. We included case reports, case series, observational studies, and literature reviews. We included patients with primary Sjogren’s syndrome with rare cardiac presentations, excluding major cardiovascular events like coronary artery disease and ischemia-related heart failure.

Results

After excluding duplicates and non-relevant articles, 27 studies with 31 patients diagnosed with only primary Sjogren's syndrome were included in this analysis (Table 1). The mean age was 42.16 years and the sex distribution was 5/31 males (16.12%) and 26/31 (83.87%) females. Common presenting symptoms included fever, chest pain, dyspnea, and syncope. Most frequently reported cardiac manifestation included atrioventricular blocks (n=12, 38.70%), autoimmune myocarditis (n=8, 25.80%), pericardial effusion (n=6, 19.35%), pericarditis (n=1, 3.22%), cardiac diffuse large B-cell lymphoma (n=2, 6.45%), and reversible cardiomyopathy (n=2, 6.45%) of the patient population. In terms of serology, 25/26 (96.15%) patients were positive for anti-Sjögren's syndrome-related antigen A autoantibodies (SSA), 18/26 (66.23%) were positive for anti-Sjögren's syndrome-related antigen B autoantibodies (SSB), 15/26 were positive for ANA (57.69%), and 6/26 (2.07%) were positive for RF.

Discussion

Primary Sjogren's syndrome is an autoimmune disease that affects 0.5-1% of the population and the most common symptoms include fever, fatigue, arthritis, Raynaud’s phenomena, and dry mucus membranes including keratoconjunctivitis sicca and xerostomia [1]. Recently, there have been studies that insinuated that there might be rare cardiac involvement associated with primary Sjogren’s syndrome. Within this literature review, we aimed to explore the reported cardiac manifestations in patients with primary Sjogren's syndrome. We studied cases with only primary Sjogren's syndrome to decrease the confounding effects of other rheumatological diseases such as SLE and RA. Although we were able to limit bias with other rheumatological diseases, the same cannot be said for the cardiac risk factors. As we know, atherosclerosis is the principal cause of mortality in up to 75% of cardiovascular diseases and can be explained by classic risk factors like obesity, smoking, and hypertension. However, evidence suggests that chronic inflammation secondary to autoimmune diseases like primary Sjogren's syndrome can independently contribute to the acceleration of the atherosclerotic process, primarily through endothelial dysfunction [2,3]. Two case-controlled studies by Lodde and Vaudo et al. have indicated that primary Sjogren's syndrome may be associated with elevated serum lipid levels and rapid atherosclerosis, potentially leading to multiple cardiac complications [4,5].

Among these manifestations, atrioventricular blocks have been identified as significant findings in this patient population. However, the association between AV nodal block and SSA/SSB antibodies, autoantibodies commonly found in primary Sjogren's syndrome, remains a subject of controversy, as discussed by Baugmart et al. in their study [6]. Autoimmune myocarditis is another cardiac complication that has been reported in patients with primary Sjogren's syndrome. Mutsukura et al. described a case of autoimmune pericarditis in a patient with primary Sjogren's syndrome, successfully treated with steroid therapy [7]. While these findings may potentially be attributed to other underlying causes, it is crucial to maintain a high clinical suspicion for cardiac complications in patients suffering from primary Sjogren's syndrome, as overlooking such manifestations can lead to severe consequences.

To further elucidate the etiology and associations between primary Sjogren's syndrome and various cardiac manifestations, additional trials and studies are warranted so that we can enhance our understanding of the cardiac implications of this autoimmune disease, and improve the outcomes for affected individuals. 

**Table 1:** Cardiac Manifestations of Primary Sjogren's Syndrome ANA: antinuclear antibody; SSA: anti–Sjogren's syndrome-related antigen A autoantibodies; SSB: anti–Sjogren's syndrome-related antigen B autoantibodies; RF: rheumatoid factor; NSAIDs: non-steroidal anti-inflammatory drugs; PPM: permanent pacemaker; AV block: atrioventricular block; ACE- I: angiotensin-converting enzyme inhibitors

Author	Year	No. of patients	Age	Sex	Presenting symptoms	Serology	Cardiac manifestation	Treatment	Outcome
Mutsukura et al [7]	2007	1	35	F	Fever, chest pain	ANA, SSA, RF	Autoimmune pericarditis	Steroids, heparin	Improved
Rajani et al [8]	2013	1	50	F	Dyspnea, palpitations	ANA, SSA, SSB, RF	Pericardial effusion	Steroids, pericardiocentesis	Improved
Shin et al [9]	2014	1	45	F	NA	ANA, SSB	Pericardial effusion	Steroids, NSAID	NA
Nayfeh et al [10]	2019	1	46	M	Fever, chest pain, dyspnea	ANA, SSA, SSB	Pericardial effusion	NSAID, colchicine	Improved
Medik et al [11]	2022	1	54	F	Fever, chest pain, dyspnea	NA	Pericardial effusion	Steroids, pericardial window, antibiotics	Improved
Abrams et al [12]	2018	1	27	F	Chest pain, dyspnea	ANA, SSA, SSB	Pericardial effusion	Steroids, epoprostenol, PDE-5 inhibitor, pericardial window	Improved
Amaechi et al [13]	2021	1	50	F	Fever, chest pain	ANA, SSA, SSB	Pericardial effusion	NSAID, colchicine	Improved
Yoong et al [14]	2007	1	84	F	Dyspnea	SSA, SSB	Cardiac lymphoma (DLBCL)	Steroids, pericardial window	Progression of DLBCL
Kino et al [15]	1995	1	NA	F	Lightheadedness	NA	Cardiac lymphoma (DLBCL)	Surgical resection, chemotherapy	Improved
Golan et al [16]	1997	1	40	F	NA	SSA	Reversible cardiomyopathy	Steroids, cyclophosphamide	Improved
Vindhyal et al [17]	2018	1	64	F	Fatigue	NA	Takotsubo cardiomyopathy	Steroids	Improved
Levin et al [18]	1999	1	48	F	Fever, nausea, dyspnea	SSA	Autoimmune myocarditis	Steroids, diuretics, ACE-I, digoxin	Improved
Llanos-Chea et al [19]	2016	1	74	F	Fever, chest pain, dyspnea	ANA, SSA, SSB, RF	Autoimmune myocarditis	Steroids	Improved
Chung et al [20]	2001	1	39	F	Fatigue, dyspnea	NA	Autoimmune myocarditis	Steroids, diuretics, ACE-I, digoxin, steroids	Improved
Yoshioka et al [21]	1999	1	68	M	Fever, fatigue	ANA, SSA, SSB, RF	Autoimmune myocarditis	Antibiotics	Improved
Watanabe et al [22]	2018	1	35	F	Fever, headache, nausea	SSA, SSB	CVAB, Autoimmune myocarditis	Steroids, IVIG	Improved
Kau et al [23]	2017	1	59	F	Fever, myalgia, edema	NA	Autoimmune myocarditis	NA	Improved
Zehlicke et al [24]	2020	1	37	F	Dyspnea, edema, arthralgias	ANA, SSA, SSB, RF	Autoimmune myocarditis	ACE-i, B-blocker, digoxin	Improved
Caballero-Güeto et al [1]	2007	1	74	F	Chest pain, fatigue, dyspnea	ANA, SSA, SSB	Autoimmune myocarditis	ACE-i, B-blocker, digoxin	Improved
Jobling et al [25]	2018	1	44	F	Dizziness, palpitations	SSA, SSB	Intermittent complete heart block	Dual chamber pacemaker	Improved
Baumgart et al [5]	1998	1	76	F	Syncope	ANA, SSA, SSB	Bradycardia with AV block	Steroids, thyroxine, hydroxychloroquine	Improved
Sung et al [26]	2011	1	49	F	Dizziness	ANA, SSA	Variable heart block	PPM	Improved
Santos Pardo et al [27]	2013	1	26	F	Syncope	ANA, SSA	Complete heart block	Steroids, azathioprine	Improved
Lodde et al [28]	2005	5	69	3M, 2F	NA	SSA, SSB	First-degree heart block	NA	NA
Lee et al [29]	1996	1	39	F	Syncope	ANA, SSA, RF	Complete heart block	PPM	Stable
Ehtesham et al [30]	2022	1	18	F	Syncope	SSA	Second-degree heart block	PPM	Improved
Tam et al [31]	2018	1	57	F	Dizziness	ANA SSA	Complete heart block	PPM	Improved

## Conclusions

Although there is significant evidence that a higher CV risk burdens SS patients, the causes and factors accounting for it are yet to be fully understood. Present data suggest that systemic vasculopathy, B-cell activation, and autoimmunity could play a role in the pathophysiology of these cardiac disorders. These manifestations, even though rare, if left untreated, can lead to increase mortality in this patient population. These findings should be the object of a careful investigation, aiming at preventing, with an early diagnosis and intervention, serious complications, including sudden cardiac death.
